# Arginase 1 Deficiency: using genetic databases as a tool to establish global prevalence

**DOI:** 10.1186/s13023-022-02226-8

**Published:** 2022-03-02

**Authors:** C. Catsburg, S. Anderson, N. Upadhyaya, M. Bechter

**Affiliations:** 1 BluePrint Orphan, New York, NY USA; 2 Aeglea BioTherapeutics, Inc., Austin, TX USA

## Abstract

**Background/objective:**

Arginase 1 Deficiency (ARG1-D) is a rare inherited metabolic disease with progressive, devastating neurological manifestations with early mortality and high unmet need. Information on prevalence is scarce and highly variable due to limited newborn screening (NBS) availability, variability of arginine levels in the first days of life, and high rates of misdiagnosis. US birth prevalence was recently estimated via indirect methods at 1.1 cases per million live births. Due to the autosomal recessive nature of ARG1-D we hypothesize that the global prevalence may be more accurately estimated using genetic population databases.

**Methods:**

MEDLINE and EMBASE were systematically searched for previously reported disease variants. Disease variants in ARG1-D were annotated wherever possible with allele frequencies from gnomAD. Ethnicity-specific prevalence was calculated using the Hardy–Weinberg equation and applied to generate country-specific carrier frequencies for 38 countries. Finally, documented consanguinity rates were applied to establish a birth prevalence for each country.

**Results:**

133 of 228 (58%) known causative alleles were annotated with ethnic-specific frequencies. Global birth prevalence for ARG1-D was estimated at 2.8 cases per million live births (country-specific estimates ranged from 0.92 to 17.5) and population prevalence to be 1.4 cases per million people (approximately 1/726,000 people). Birth prevalence estimates were dependent on population demographics and consanguinity rate.

**Conclusion:**

Birth prevalence of ARG1-D based on genetic database analysis was estimated to be more frequent than previous NBS studies have indicated. There was a higher degree of confidence in North American and European countries due to availability of genetic databases and mutational analysis versus other regions. These findings suggest the need for greater disease education around signs and manifestations of ARG1-D, as well as more widespread testing and standardization of screening for this severe disease in order to appropriately identify patients prior to disease progression.

**Supplementary Information:**

The online version contains supplementary material available at 10.1186/s13023-022-02226-8.

## Introduction

Arginase 1 Deficiency (ARG1-D) is a debilitating and progressive metabolic disease characterized by persistent elevation of arginine and its metabolites, ultimately leading to significant morbidity and early mortality [1, 2]. It is one of a group of urea cycle disorders (UCDs) presenting in childhood with manifestations that include lower-limb muscle weakness, spasticity, developmental and motor delays, intellectual disability, and seizures [3]. Spasticity is the most overt sign and differentiates ARG1-D from other UCDs. Preliminary symptoms also include irritability, vomiting and feeding issues, and failure to thrive [1]. Hyperammonemia can occur but is usually less frequent and less severe when compared to other UCDs [4] .

ARG1-D is currently estimated to account for approximately 3.5% of all UCD cases but estimates of disease prevalence are inconsistent [5]. Previous reports of incidence vary by an order of magnitude, from 0.5 to 5.0 cases per million [6, 7]. US birth prevalence of ARG1-D was recently estimated via indirect methods at 1.1 cases per million live births [8]. A US-based newborn screening (NBS) study predicted a similar rate, estimating birth prevalence at 0.9 cases per million live births [9]. However, estimating prevalence of ARG1-D via NBS can be challenging due to the variability of arginine levels in the first days of life and since ARG1-D is not universally included on all NBS panels [10]. Arginine accumulates slowly after birth and as a result the range of levels in affected and unaffected newborns can overlap, making it difficult to determine an appropriate diagnostic threshold to accurately assess arginine deficiency in neonates [9]. For this reason, patients can often be missed at the newborn stage and these studies could underestimate the true prevalence of ARG1-D. There is also published evidence that ARG1-D patients are misdiagnosed as suffering from more recognized disorders such as hereditary spastic paraplegia (HSP) and cerebral palsy (CP) [11].

In the neonate, given the complications of diagnosis via arginine levels, genetic diagnosis of ARG1-D is the most reliable method of determining a positive case of the disease [3]. More than 60 causative *ARG1* genetic variants have been identified to date [12]. Due to the autosomal recessive inheritance of ARG1-D, we hypothesize that the global prevalence of the disease may be more accurately estimated via genetic population databases, such as seen in other autosomal recessive diseases [13]. However, birth prevalence based on carrier frequency assumes random mating, an assumption which is violated in the event of consanguineous marriage. Birth defects in consanguineous marriages are estimated to be 2–3% higher compared to the general population, and lead to higher rates of autosomal recessive disorders [14].

Today, the primary treatment of ARG1-D is elimination of protein from the diet to minimize arginine intake, with EAA supplementation to maintain nutritional status; use of nitrogen scavengers is also commonplace [3]. Early intervention is critical to slow disease progression, so understanding the true birth prevalence of the disease is becoming increasingly important, especially as evidence of misdiagnosis builds. This study aims to estimate the global genetic prevalence of ARG1-D via genetic population databases and is the first study to adjust for consanguinity within these methods.

## Materials and methods

### Search strategy

To evaluate the full genetic spectrum of ARG1-D, a systematic literature review was performed using MEDLINE (PubMed) and EMBASE (Scopus). The following search criteria terms were used: ‘ARG1 AND deficiency’; ‘(arginase 1) AND deficiency’; ‘ARG1 AND mutation’; ‘(arginase 1) AND mutation’. Abstracts of all studies identified via this search were first screened by two independent reviewers (S.A. and C.C.) to identify potentially relevant articles reporting information on ARG1-D cases. Publications that met the inclusion criteria at the abstract stage were flagged for full-text review, and any disagreements were resolved through discussion. Studies were then thoroughly reviewed (S.A. and C.C.) for reports of any ARG1-D cases with verified genetic information. Reference lists of articles selected for full-text review were also searched to identify additional publications that may report on ARG1-D cases. The search was not limited by date or geographical region. All diagnosed ARG1-D cases identified with at least one known causative genetic mutation were recorded into a database. Care was taken to deduplicate cases that were reported more than once in the literature.

### Frequency annotation via gnomAD

A list of all known pathogenic *ARG1* variants currently identified as causative for ARG1-D, including the frequency of each within ARG1-D cases, was compiled from the case database referenced above. Each reported mutation was standardized and matched to its Reference SNP cluster ID (rsID) number (Additional file 1: Table S1) [15]. Subsequently, each mutation identified was queried via rsID number in the gnomAD database (v2) [16]. Where possible, *ARG1* variants were annotated with population frequencies obtained from the gnomAD database for the following ethnic groups: African/African-American (AFR), Latino/Admixed American (AMR), East Asian (EAS), European (non-Finnish) (NFE) and South Asian (SAS). These ethnic groups are the most extensively genotyped and catalogued in the gnomAD database [16]. None of the pathogenic variants were found in the Finnish population so the FIN population were excluded due to a small non-representative sample size. Once the carrier frequency for each variant identified in the case database was obtained, the frequencies across all pathogenic variants were summed within each ethnic sub-group to obtain overall carrier frequencies for *ARG1* by ethnicity (Table 1). Ninety-five percent confidence intervals (CIs) for these estimates were calculated using the binomial (Clopper-Pearson) "exact" method based on the beta distribution. Because census data used in this study does not always distinguish between Asian sub-groups, the EAS and SAS populations were then combined into a single Asian population to enable analysis.

**Table 1:** *ARG1* mutation carrier frequency in each of the four race groups

SNP Name	Allele patient count	NFE	AFR	AMR	EAS/SAS
rs104893944	28	0.000031	0.000000	0.000339	0.000000
rs104893948	21	0.000016	0.000000	0.000113	0.000050
rs587776539	12	0.000018	0.000000	0.000000	0.000000
rs140549609	8	0.000009	0.000000	0.000000	0.000000
rs753829097	7	0.000009	0.000000	0.000000	0.000000
rs28941474	7	0.000000	0.000000	0.000029	0.000000
rs377280518	6	0.000040	0.000080	0.000000	0.000000
Other^a^	139	0.000742	0.000106	0.000923	0.001178
**Total**	**228**	**0.000865**	**0.000186**	**0.001404**	**0.001228**

^a^Other is comprised of all mutations that occurred less than 5 times in the 228 alleles

### Birth prevalence and population prevalence estimates

Using the identified *ARG1* carrier frequencies by ethnic sub-group from the case database, country-specific birth and population ARG1-D prevalence estimates were then generated. This analysis focused on 38 countries that together account for 23.3% of the world population (Table 2) [17]. These countries were selected due to the availability of census data needed to estimate the ethnic breakdown, and to provide a representative sampling of the global population. For each of the 38 countries, the population percentage breakdown across the four main ethnic groups referenced above was estimated using census data where available [18–29], as well as other sources [30, 31]. For each country, the countrywide percentage of each ethnic group was multiplied by the *ARG1* carrier frequency in that group and then summed to obtain baseline country-specific *ARG1* mutation carrier frequencies (Table 2). Next, a baseline birth prevalence was calculated for each country assuming complete autosomal recessive inheritance and random mating according to the Hardy–Weinberg equilibrium. Affected individuals need two copies of a mutation to develop disease, thus the expected case frequency was equal to the carrier frequency squared. To adjust for consanguinity, consanguinity estimates for each of the 38 countries were obtained [32, 33]. Consanguinity adjustments were conservative; the percentage of consanguineous marriage that were first cousins or closer was used and more distant relationships were ignored. Consanguinity-adjusted birth prevalence estimates were calculated using the following equation:

$$(1 - f)p + f\left( {{\raise0.7ex\hbox{$1$} \!\mathord{\left/ {\vphantom {1 {16}}}\right.\kern-\nulldelimiterspace} \!\lower0.7ex\hbox{${16}$}}\sqrt f + {\raise0.7ex\hbox{${15}$} \!\mathord{\left/ {\vphantom {{15} {16}}}\right.\kern-\nulldelimiterspace} \!\lower0.7ex\hbox{${16}$}}f} \right)$$

where $f$ is equal to carrier frequency and $p$ is equal to birth prevalence.

**Table 2:** Calculated ARG1-D birth prevalence by country

Country	*ARG1* carrier frequency	ARG1-D baseline birth prevalence (births/million)	ARG1-D adjusted birth prevalence^a^ (95% CI)
Argentina	0.0017	2.85	3.06 (2.11–4.56)
Australia	0.0010	0.99	1.11 (0.87–1.55)
Austria	0.0009	0.90	1.19 (0.98–1.53)
Bahrain	0.0011	1.27	6.05 (4.78–8.23)
Belgium	0.0009	0.87	1.46 (1.23–1.84)
Brazil	0.0013	1.81	2.90 (2.19–4.02)
Canada	0.0010	1.00	1.94 (1.55–2.62)
Chile	0.0016	2.62	3.33 (2.37–4.82)
Colombia	0.0016	2.64	3.76 (2.70–5.41)
Croatia	0.0009	0.88	0.94 (0.76–1.23)
Czech Republic	0.0009	0.82	0.93 (0.76–1.25)
Denmark	0.0009	0.89	1.59 (1.35–1.97)
Finland	0.0009	0.90	0.96 (0.77–1.27)
France	0.0010	1.03	1.54 (1.24–2.04)
Germany	0.0009	0.86	1.27 (1.06–1.62)
Ireland	0.0009	0.89	1.19 (0.97–1.55)
Israel	0.0010	0.92	7.79 (6.80–9.36)
Italy	0.0010	0.91	1.21 (0.99–1.56)
Japan	0.0013	1.69	4.86 (3.46–7.43)
South Korea	0.0013	1.70	1.70 (1.02–3.15)
Kuwait	0.0011	1.21	12.97 (10.58–16.92)
Mexico	0.0016	2.55	2.85 (1.99–4.20)
Netherlands	0.0010	0.91	1.03 (0.82–1.38)
New Zealand	0.0010	0.93	1.05 (0.82–1.49)
Norway	0.0009	0.87	0.92 (0.75–1.22)
Poland	0.0009	0.88	1.17 (0.97–1.50)
Portugal	0.0010	0.91	1.81 (1.53–2.24)
Qatar	0.0012	1.35	17.48 (13.95–23.28)
Russian Federation	0.0009	0.88	1.29 (1.08–1.63)
Saudi Arabia	0.0010	0.95	14.63 (12.79–17.48)
Spain	0.0010	0.96	3.47 (2.98–4.22)
Sweden	0.0009	0.85	1.20 (1.00–1.54)
Switzerland	0.0009	0.86	1.21 (1.01–1.56)
Taiwan	0.0013	1.70	3.17 (2.15–5.14)
Turkey	0.0009	0.89	6.32 (5.59–7.39)
United Arab Emirates	0.0012	1.33	12.81 (10.09–17.44)
United Kingdom	0.0010	0.91	1.15 (0.92–1.55)
United States	0.0012	1.35	1.50 (1.09–2.20)

^a^Adjusted for estimated consanguinity rates within each country

Finally, to estimate overall country-specific prevalence, the adjusted birth prevalence rates were converted to represent population prevalence by assuming an average life expectancy for ARG1-D of 40 years.

## Results

### Study selection

**Fig. 1:**
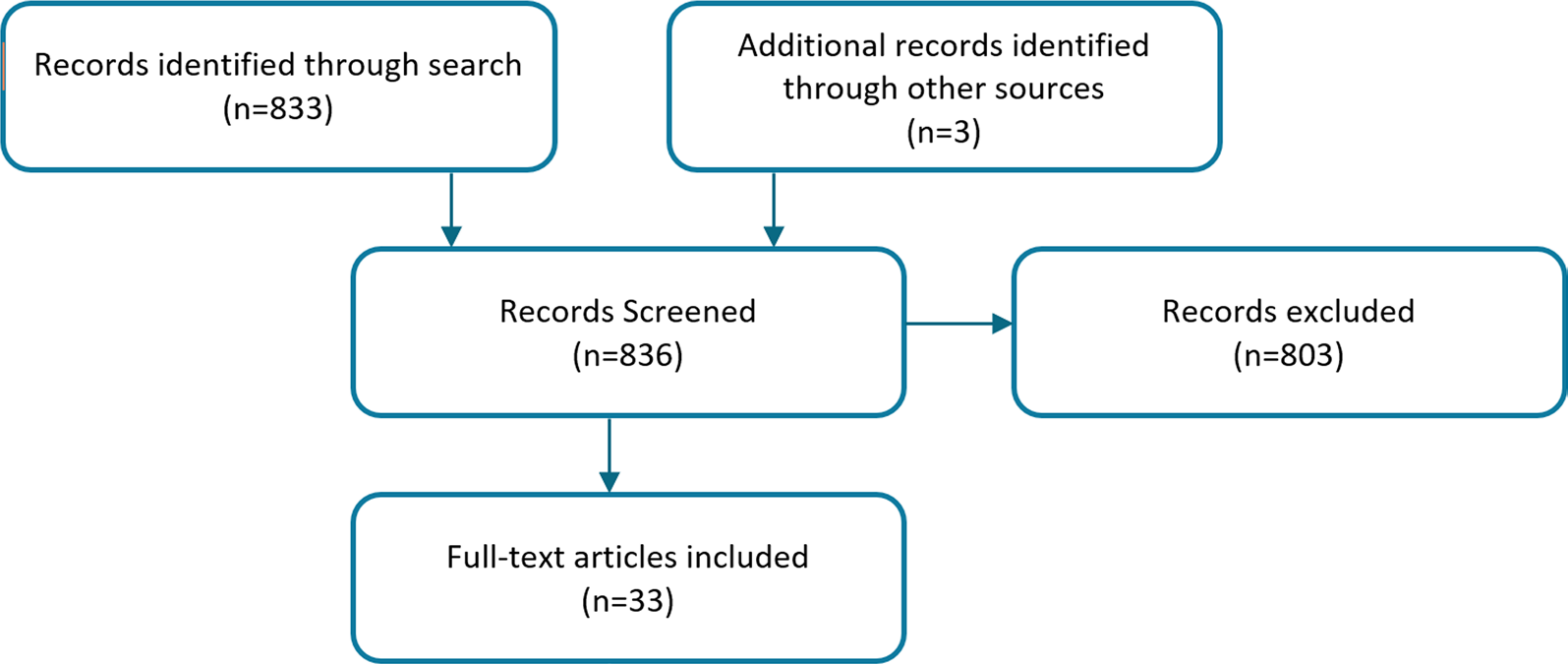
Search strategy and flow chart of study selection. Inclusion criteria are listed in methods

A total of 836 articles were identified in the primary literature search (Fig. 1). Of these, 762 studies were excluded during first-stage screening if the title/abstract clearly indicated that no mutation data on ARG1-D cases were reported. Of the remaining 74 studies, 41 were excluded after full assessment by the reviewers. Finally, a total of 33 unique publications containing genetic information on 114 total ARG1-D cases were retrieved. These 114 cases, along with their complete genetic information (where available), were entered into the database.

### Full genetic spectrum for ARG1-D

A total of 62 unique mutations responsible for ARG1-D were identified in the 114 cases found in the literature. GnomAD allele frequency data were available for 28/62 (45.2%) of these variants (Additional file 1: Table S1). 28 identified mutations accounted for 133 of the 228 (58.3%) total alleles found in the 114 ARG1-D cases (Table 1). Each mutation identified in the gnomAD database was annotated with carrier frequencies for each of the five ethnic groups (NFE, AFR, AMR, EAS, SAS). After summing the 28 mutation frequencies across the ethnic groups, the results were adjusted (divided by 58.3%) to account for the unknown alleles. This resulted in birth prevalence frequencies for the differing ethnic groups as follows—NFE: 0.75 cases per million live births; AFR: 1.30 cases per million live births; AMR: 1.97 cases per million live births; EAS/SAS: 1.51 cases per million live births. Thus, ARG1-D is more likely to occur in Latino populations, followed by Asian and African populations, and least likely in European populations.

### Country-specific birth and population prevalence estimates

Country-specific birth prevalence estimates accounting for ethnic-specific carrier frequencies are presented in Table 2. Prior to adjusting for consanguinity, the Latin countries had the highest birth prevalence estimates owing to the increased carrier frequency in the Latino/Admixed American (AMR) ethnic group. Unadjusted for consanguinity, the country with the highest ARG1-D birth prevalence is Argentina with 2.9 cases per million live births, closely followed by Colombia, Chile and Mexico, all with a birth prevalence of 2.6 per million live births.

After adjusting for consanguinity, it became apparent that interfamily marriage is the main driver behind the four countries with the highest birth prevalence of this disease (Table 2). The most prevalent countries are all from the Middle East as follows: Qatar (17.5 per million live births), Saudi Arabia (14.6 per million live births), Kuwait (13.0 per million live births), and United Arab Emirates (12.8 per million live births). Countries with homogeneous white European populations and very low consanguinity such as Norway, Finland, and the Netherlands, have the lowest estimates, each with an adjusted ARG1-D birth prevalence of 0.5 per million live births. The overall global birth prevalence of ARG1-D across all 38 countries was calculated at 2.8 cases per million live births.

A total of 2511 total cases of ARG1-D are estimated across the 38 countries investigated, resulting in an overall prevalence of 1.38 cases per million population (Table 3). Although this analysis only considered 38 countries, a racially and geographically diverse selection of countries was used, and thus these results are expected to reflect the true global prevalence. Similar to birth prevalence, the highest population prevalence estimates are seen in countries with high consanguinity (Qatar, Kuwait, UAE and Saudi Arabia) where estimates ranged from 6.4 to 8.7 cases per million population. Birth prevalence also has a high range in countries with a higher proportion of Latin population, such as Argentina, Brazil, Chile, and Colombia, with estimates within the range of 2.9 to 3.8 cases per million population. Similarly to birth prevalence, countries with predominantly homogeneous white European populations and very low consanguinity have the lowest population prevalence of ARG1-D.

**Table 3:** Calculated ARG1-D case number and population prevalence by country

Country	Population 2021 (1000 s)	Estimated ARG1-D cases	ARG1-D population prevalence (cases/million)
Argentina	45,914	70	1.53
Australia	25,700	14	0.56
Austria	8801	5	0.60
Bahrain	1744	5	3.02
Belgium	11,669	9	0.73
Brazil	215,278	312	1.45
Canada	37,924	37	0.97
Chile	18,605	31	1.67
Colombia	50,576	95	1.88
Croatia	4092	2	0.47
Czech Republic	10,634	5	0.47
Denmark	5819	5	0.80
Finland	5599	3	0.48
France	65,955	51	0.77
Germany	82,591	52	0.64
Ireland	4926	3	0.59
Israel	8842	34	3.90
Italy	59,038	36	0.61
Japan	126,109	306	2.43
South Korea	51,667	44	0.85
Kuwait	4361	28	6.49
Mexico	135,384	193	1.43
Netherlands	17,230	9	0.51
New Zealand	4876	3	0.52
Norway	5500	3	0.46
Poland	37,847	22	0.59
Portugal	10,183	9	0.90
Qatar	2840	25	8.74
Russia	143,637	93	0.65
Saudi Arabia	35,263	258	7.32
Spain	46,450	81	1.73
Sweden	10,188	6	0.60
Switzerland	8731	5	0.60
Taiwan	23,873	38	1.58
Turkey	84,515	267	3.16
UAE	9937	64	6.40
UK	67,699	39	0.58
United States	333,783	250	0.75
**Total (38 countries)**	**1,823,780**	**2511**	**1.38**

## Discussion

This report presents the findings of a genetic analysis that estimated global birth prevalence for ARG1-D to be 2.8 cases per million live births (1/357,000 live births) and population prevalence to be 1.4 cases per million people (approximately 1/726,000 people). Prevalence estimates from this genetic analysis are higher than reported in most previous studies.

Screening for ARG1-D is conducted by measuring the elevation of arginine in newborn screening blood spots via tandem mass spectrometry, and is currently included as a secondary target on the US Recommended Uniform Screening Panel [9]. A US newborn screening study published in 2017 reported a birth prevalence of approximately 0.9 per million live births [9]. A separate study published in 2013 using both newborn screening data and data from the Urea Cycle Disorders Consortium (UCDC) found a similar ARG1-D birth prevalence at 1.1 per million live births (1/950,000 live births) [8]. The estimate for birth prevalence presented in this study indicate the US birth prevalence of ARG1-D at 1.5 cases per million live births, approximately 50% higher than these studies report. Newborn screening studies are often considered the gold standard of birth prevalence estimates; however, with ARG1-D in particular, screening can be challenging as there is variability in arginine levels in the first days of life [10]. Moreover, the appropriate arginine level to diagnose a case at birth has been difficult to determine due to the overlap between arginine levels in affected and unaffected newborns. The cutoff for a positive screening result also varies across states, further complicating the calculation of birth prevalence estimates from newborn screening studies [9]. The higher genetic analysis estimates presented in this study suggest that the newborn screenings are currently under-capturing potential cases.

Estimating prevalence via genetic databases is reliant on the amount and precision of currently available information. In this study, ethnic-specific frequencies were annotated from 133 of 228 (58%) causative ARG1-D alleles. While the most common mutations are captured here, uncertainty remains regarding the remaining 42% of alleles. Over time it is expected that it will be possible to refine and improve these estimates as more sample genomes and information on more genetic variants are added to databases such as gnomAD. However, adjusting the currently annotated mutations by the percentage of unknown variants currently existing in cases should result in a very good approximation of the true prevalence. When estimating the global prevalence and birth prevalence of ARG1-D, 38 countries that had the most available information on racial breakdown were included, predominantly using census data. While these countries make up nearly 25% of the global population, we have not yet estimated prevalence in every country globally. However, since the countries represented here are geographically varied and racially diverse, the reported estimates in this study should be an appropriate representation of a global estimate. To the best of the authors’ knowledge, this is the first study to adjust prevalence estimates based on carrier frequency for consanguinity in each country. It has been established that high consanguinity (such as seen in many Arab countries) leads to a sharp increase in the birth rate of autosomal recessive disease [32, 34]. Thus, adjusting for this factor provides a more accurate reflection of the current state of ARG1-D birth prevalence globally. This can be seen reflected in the study case database; for example, within European ARG1-D cases, patients with a Turkish origin where consanguinity is higher than the rest of Europe are much more represented than we would expect based on genetic carrier frequency alone.

A potential limitation of this study is that assumptions of consanguinity and life expectancy may not be generalizable to all populations. Consanguinity in the current study was limited to marriage of first cousins or closer, and rates of consanguinity, even using this conservative definition, are likely higher in reality than estimated here. Further, although an estimated median life expectancy of 40 years was selected based on the literature and clinical experience, actual life expectancy is likely lower in developing countries and regions where disease awareness, access to diagnostic procedures, and implementation of management strategies remain a challenge.

The findings from this present study suggest that ARG1-D may be more prevalent than previously thought, indicating a lack of diagnosis or misdiagnosis. Awareness of ARG1-D and the burden it brings to patients, caregivers and healthcare systems, may minimize delays in diagnosis which are otherwise associated with poor outcomes. Diagnosis of ARG1-D is possible through simple amino acid testing (since elevated levels of arginine are seen in the majority of patients) and can be confirmed with genetic testing or analysis of red blood cell arginase levels. The utility of this analysis is to highlight that ARG1-D disease prevalence may be currently underestimated, because of poor disease awareness. Greater awareness to better recognize the signs and symptoms of ARG1-D, as well as more widespread and standardized screening, is needed to ensure proper and timely diagnosis of this severe disease.

## Supplementary Information

**Additional file 1.** Each *ARG1* variant reported among the 114 cases in the literature was standardized and matched to its Reference SNP cluster ID (rsID) and queried in the gnomAD database. GnomAD allele frequency data were available for 28 of 68 of these variants (45%).

## Data Availability

The datasets generated during and/or analyzed during the current study are available in GnomAD, https://gnomad.broadinstitute.org/
