# Supplementary material for: Arginase 1 Deficiency: using genetic databases as a tool to establish global prevalence
Source: Orphanet J Rare Dis. 2022 Mar 2;17:94. doi: 10.1186/s13023-022-02226-8 (PMC8889696; doi:10.1186/s13023-022-02226-8)
Supplement: Supplementary file 1 — Additional file 1. Each ARG1 variant reported among the 114 cases in the literature was standardized and matched to its Reference SNP cluster ID (rsID) and queried in the gnomAD database. GnomAD allele frequency data were available for 28 of 68 of these variants (45%). [file 13023_2022_2226_MOESM1_ESM.docx]

| **Table S1. Identified causative mutations in ARG1-D found in gnomAD databases** | | |
| --- | --- | --- |
| rsID number | Protein Change | DNA Change |
| rs104893944 | p.Arg21Ter | c.61C>G |
| rs104893948 | p.Gly235Arg | c.703G>A |
| rs587776539 | splice donor | c.57+1G>A |
| rs140549609 | p.Asp128Gly | c.383A>G |
| rs753829097 | p.Gly99Arg | c.295G>A |
| rs28941474 | p.Ile11Thr | c.32T>C |
| rs377280518 | p.Arg308Gln | c.923G>A |
| rs755975244 | intron variant | c.93del |
| rs149310631 | p.Ile8Thr | c.23T>C |
| rs104893940 | p.Arg291Ter | c.871C>T |
| rs104893947 | p.Trp122Ter | c.365G>A |
| rs756080885 | p.Leu216 Alafs*4 | c.646_649delCTCA |
| rs1554250849 | p.His141Leu | c.422A>T |
| rs1554249332 | P.Met1Thr | c.2T>C |
| rs1464239857 | p.Arg225Glyfs*5 | c.673delA |
| rs1422422629 | p.Asn69Ile | c.206A>T |
| rs1356179382 | p.Gly18Glu | c.53G>A |
| rs1326930389 | p.Gly27Asp | c.80G>A |
| rs755975246 | p.Arg32Glufs*16 | c.93delG |
| rs755975245 | intron variant | g.183827881G>T |
| rs1554251045 | splice acceptor | c.466-2A>G |
| rs767219084 | p.Gly142Glu | c.425G>A |
| rs757959356 | p.Arg71Thr | c.212G>C |
| rs755359126 | p.Ala298Pro | c.892G>C |
| rs104893943 | p.Gly138Val | c.413G>T |
| rs866970619 | p.Gly305Arg | c.913G>A |
| rs748744950 | p.Glu42Ter | c.124G>T |
